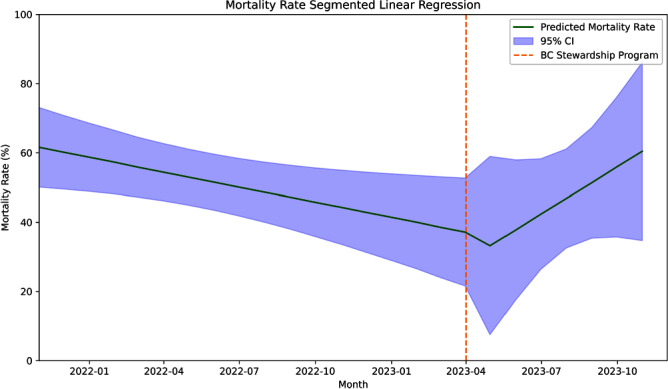# Reduced Blood Culture Use in High-Volume Lung Transplant MICU Following a Stewardship Program, Northern California, 2021–2023

**DOI:** 10.1017/ash.2024.223

**Published:** 2024-09-16

**Authors:** Guillermo Rodriguez Nava, Javier Lorenzo, Mindy Sampson, Valeria Fabre, Sara Cosgrove, Jorge Salinas

**Affiliations:** Stanford University School of Medicine; Stanford University; Johns Hopkins University School of Medicine

## Abstract

**Introduction:** There is a need to optimize blood culture (BC) utilization among hospitalized adults. A previous study showed an evidence-based BC algorithm improved BC utilization in a medical intensive care unit (MICU) at a large academic center. Our aim is to evaluate the impact of an intervention based on this algorithm on blood culture utilization in the MICU at Stanford Health Care, a referral center known for its high volume of solid organ transplants. **Methods:** We conducted a before-after study evaluating the impact of a BC diagnostic stewardship program in the MICU at Stanford Health Care, a 20-bed unit with an average of 20% lung transplant patients per day. All patients ≥18 years of age admitted to the unit during the study period were included. We adopted a previously published evidence-based algorithm detailing syndromes with low and high risk for bacteremia, which was referenced during patient rounds. Additionally, education and feedback to providers about BC utilization and indication inappropriateness was performed during leadership meetings every other month. We performed an interrupted time series analysis using historical data 17 months before the implementation of the blood culture stewardship program on April 1, 2022 and 8 months after. We assessed changes on BC utilization adjusted to patient days and crude mortality rate during the same period as a balancing measure. **Results:** Before the implementation of the program, the median BC utilization was 216 per 1,000 patient days (range 150–250 per 1,000 patient days). Following the introduction of the program, there was a significant decrease in blood culture utilization by 5% (95% CI -10% to -1%, p=.02; Figure 1). Post-intervention, the blood culture stabilized, with no significant increase observed (0.09% increase, 95% CI -0.7% to 1%, p=.81). The mortality rate, prior to the implementation of the program, had shown a significant downward trend of 1.4% over time (95% CI -3% to -0.2%, p=.02). After the intervention, a nonsignificant decrease of 8% was observed (95% CI -44% to 27%, p=.62; Figure 2), followed by a nonsignificant upward trend of 6% (95% CI -1% to 13%, p=.10). **Conclusion:** We observed a significant reduction in BC utilization after implementing the BC diagnostic stewardship program in a MICU frequented by a high number of lung transplant patients.

**Figure:**
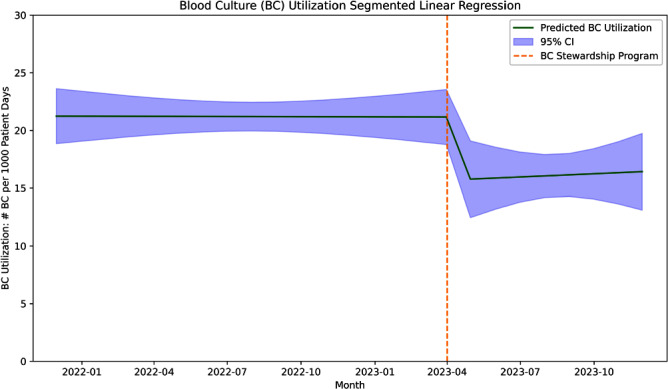

**Figure:**